# A Genotype-Independent, Simple, Effective and Efficient in Planta *Agrobacterium*-Mediated Genetic Transformation Protocol

**DOI:** 10.3390/mps5050069

**Published:** 2022-09-03

**Authors:** Pushpa Kharb, Rinku Chaudhary, Narendra Tuteja, Prashant Kaushik

**Affiliations:** 1 Department of Molecular Biology, Biotechnology and Bioinformatics College of Basic Sciences and Humanities, CCSHAU, Hisar 125004, India; rinkupilania033@gmail.com; 2 Plant Molecular Biology Group, International Centre for Genetic Engineering & Biotechnology (ICGEB), Aruna Asaf Ali Marg, New Delhi 110067, India; narendratuteja@gmail.com; 3 Kikugawa Research Station, Yokohama Ueki, 2265, Kikugawa 439-0031, Japan

**Keywords:** genetic transformation, genotype-independent, protocol, *Agrobacterium*-mediated, crop improvement

## Abstract

Crop improvement under changing climatic conditions is required to feed the growing global population. The development of transgenic crops is an attractive and conceivably the most effective approach for crop improvement with desired traits in varying climatic situations. Here, we describe a simple, efficient and robust in planta *Agrobacterium*-mediated genetic transformation method that can be used in most crops, including rice, wheat and cotton, and particularly in tissue culture recalcitrant crops, such as chickpea and pigeon pea. The protocol was successfully used for the development of transgenic chickpea and pigeon pea lines for resistance against pod borer. Transgenic lines in chickpea, pigeon pea and wheat were also developed for salt stress tolerance. These lines exhibited improved salt tolerance in terms of various physio-biochemical parameters studied. Since the protocol is rapid, as no tissue culture step is involved, it will significantly contribute to the improvement of most crops and will be of interest for plant biologists working with genetic engineering or genome editing.

## 1. Introduction

Genetic transformation in plants may be defined as the integration of exogenous genes into cells, tissues or organs of flora of interest through tools of molecular and cellular biology while simultaneously enhancing the understanding of the plant physiology and playing a vital role in crop improvement [1]. A successful plant transformation is dependent on the insertion of the transgene with precision and stability, the regeneration of the transformed plant cells and the production of the non-chimeric transgenic plant [1].

Plant transformation studies employ electroporation or biolistics or *Agrobacterium*-mediated approaches to integrate exogenous genes into plant cells. While electroporation is limited by the ability of the protoplasts to develop into whole plants, biolistics is a desirable method for transformation studies associated with multiple genes or high-molecular-weight DNA [1,2]. Most importantly, these transformation methods are based on tissue culture techniques that are often associated with somaclonal variations which influence the traits of the plants [3]. These methods were observed to be limited in lieu of crops that are agronomically essential wherein the transformation efficiency could not be successfully demonstrated in vitro.

A direct, fastidious and tissue culture-independent transformation system was achieved by Feldmann and Marks wherein plants were transformed using *Agrobacterium tumefaciens*, a soil bacterium, as a biological vector of transformation, termed in planta transformation [4]. The regeneration of high numbers of transformed progeny in a short time span, cost effectiveness and minimal reagent usage render this method desirable in agricultural research, specifically towards the attainment of sustenance in crop production [5,6,7,8,9].

In planta transformation requires the incorporation of the gene of interest into the biological vector *Agrobacterium*, followed by incubation of plant parts (of the plant under study) in the recombinant bacterial culture to bring about transformation.

Various approaches for achieving *Agrobacterium*-mediated in planta transformation include floral dip, vacuum infiltration, fruit injection, apical meristem injury, pollen tube and sonication. Transformations with these methods have been successfully demonstrated with *Arabidopsis thaliana*; cereals such as wheat, rice, maize, etc.; vegetable crops of pigeon pea, chick pea, pepper, etc.; and in oil seeds [3]. While direct and efficient transformation has been achieved with these methods, the frequency of transformation, however, is low [3,6,7,8,9]. Among the various procedures of in planta transformation, the floral dip method was observed to be incompatible with plants other than *Arabidopsis*. The pollen tube method was reported to be non-reproducible despite the development of transgenic plants, and mostly depends on the technique and species. In the apical meristem injury method, the germinating seedlings were pricked with needles coated with the *Agrobacterium* culture on the apical and inter-cotyledonary regions. Transformation efficiency via this method was observed to be low. Embryonal segments and germinating seeds of pigeon pea were induced with pest resistance using *Agrobacterium*-mediated transformation followed by tissue culture before transferring to a greenhouse [10,11,12,13,14]

Therefore, the development of a robust and efficient transformation protocol that can be applied in various crops is of utmost importance.

## 2. General Procedure of the Protocol

### 2.1. Plant Material

Dry and mature seeds of the crop to be genetically modified.

### 2.2. Vector for Transformation

*Agrobacterium tumefaciens* strain LBA4404/EHA105 harboring the transgene to be transferred.

### 2.3. Preparation of Agrobacterium Culture for the Transformation

#### 2.3.1. Inoculum Preparation from Stock Culture (Day 1) • Timing 24 h

1. Streak the stock culture initially on LB agar plates containing the appropriate antibiotic(s).
2. Incubate the plates overnight at 28 °C(?) (TROUBLESHOOTING, Table 1).

#### 2.3.2. Inoculum Culture for Further Studies (Day 2) • Timing 24 h

1. Prepare the inoculum culture for further studies in LB broth wherein a single colony from the LB agar plate is inoculated in 10 mL LB broth containing the appropriate antibiotic(s).
2. Incubate LB broth with the *Agrobacterium* inoculum at 28 °C and 120 rpm in an orbital shaking incubator overnight.
3. Record O.D. of the culture at a wavelength of 600 nm(?) (TROUBLESHOOTING).

### 2.4. Agrobacterium-Mediated Transformation (Day 3)

1. Surface sterilize the seeds with 0.1% HgCl_2_ solution for 10 min. Wash the seeds with sterilized distilled water multiple times to eliminate any traces of mercuric chloride. • Timing: 10–15 min.
2. After sterilization and washing, incubate the seeds in the *Agrobacterium* culture (O.D. = 0.6) overnight in an orbital shaker at 100 rpm. • Timing: 24 h.
3. Wash the incubated seeds with distilled water and transfer to potted soil(?) (TROUBLESHOOTING).

### 2.5. Screening of Putative Transgenic Plants for Presence of the Transgene

#### Isolation of Genomic DNA from Putative Transgenic Plants (2 Days)

Isolate genomic DNA from young leaves of the putative transformed plants raised from seeds incubated with *Agrobacterium*(?) (TROUBLESHOOTING).

### 2.6. Screening of Putative Transgenic Plants for Presence of the Transgene

#### 2.6.1. Screening by PCR

Screen the putative transgenic plants by subjecting the isolated genomic DNA to PCR amplification using transgene-specific primers under appropriate conditions.

Use isolated plasmid DNA as the positive control and genomic DNA isolated from non-transformed wild-type plants (WT) as the negative control(?) (TROUBLESHOOTING).

#### 2.6.2. Screening of Putative Transgenic Plants by Expression of the Reporter Gene

For example, detection of histochemical expression of β-glucuronidase (GUS) activity in plant tissues in the putative transgenic plants by following the method of [15].

### 2.7. Detection of Integration and Copy Number of the Transgene in Transgenic Plants (3 Days)

#### 2.7.1. Perform Southern Hybridization for Detection of Integration and Copy Number of the Transgene in the T_0_ Transgenic Plants Using the Approach Given in [16]

Employ the plasmid DNA as the positive control, and genomic DNA extracted from non-genetically modified (WT) plants as the negative control.

#### 2.7.2. Determination of Transgene Copy Number Using Real-Time PCR

The transgene copy number in transgenic plants can be determined by quantitative real-time PCR also using transgene-specific primers [16]. Use WT plant as a negative control.

### 2.8. Evaluation of Transgene Efficacy in Transgenic Plants

Perform appropriate tests to check the efficacy of the transgene in the transgenic plants in the T_0_ generation. The tests can be performed in all generations raised subsequently, e.g., performing insect bioassay to detect insect resistance, etc.

## 3. Results

In this section, we present the examples where we successfully utilized the above protocol for developing transgenic lines in several crops.

### 3.1. Plant Material

Dry and mature seeds of chickpea cv. C-235, HC-1; pigeon pea cv. Manak, wheat cv. WH1184, WH1105; and indica rice cv. 1121 were used for the development of insect-resistant chick pea and pigeon pea lines, as well as for the development of salt-tolerant chickpea, pigeon pea and wheat lines (Table 2).

### 3.2. Vector for Transformation

The *Agrobacterium* strains harboring different transgenes used for testing the protocol are listed in Table 2.

### 3.3. Preparation of Agrobacterium Culture for the Transformation

#### 3.3.1. Inoculum Preparation from Stock Culture

The *A. tumefaciens* strain EHA105 harboring pBINIAa_3_ was spread on AB minimal medium agar plates supplemented with kanamycin (50 mg L^−1^) and rifampicin (10 mg L^−1^). The *A. tumefaciens* strain LBA4404 harboring pBinAR-35S*cry1Ac* was spread on LB minimal medium agar plates supplemented with kanamycin (50 mg L^−1^) and rifampicin (10 mg L^−1^). The plates were incubated overnight at 28 °C.

The *A. tumefaciens* strain LBA4404 harboring pCAMBIA 1301-*OsRuv*B/*OsLec-RLK* and the *A. tumefaciens* strain LBA4404 harboring pCAMBIA1300-*Psp68* from the glycerol stock culture were spread on LB medium agar plates supplemented with kanamycin (50 mg L^−1^), streptomycin (50 mg L^−1^) and rifampicin (50 mg L^−1^) and incubated at 28 °C for raising fresh bacterial cultures.

#### 3.3.2. Inoculum Culture for Further Studies

A single colony from freshly grown cultures was inoculated in 100 mL liquid AB minimal medium/LB medium supplemented with appropriate antibiotics and grown overnight at 28 °C on a shaker at 120 rpm. O.D. of the culture was recorded at 600 nm.

### 3.4. Agrobacterium-Mediated Transformation

For developing insect-resistant chickpea lines, seeds of chickpea cv. 235 were sterilized in 0.1% mercuric chloride solution for 10 min and then washed with sterilized distilled water 5–6 times to remove traces of mercuric chloride. The surface-sterilized seeds were soaked in the above bacterial culture with O.D. 0.6 and kept overnight in a shaker. These seeds were germinated on germination medium (20 g agar in one liter of water) containing 250 mg/L cefotaxime. Fifteen-day-old seedlings were transferred to potted soil. This method was patented [27].

For developing other transgenic lines mentioned in Table 2 [17,18,19,20,21,22,23,24,25,26], the seeds were directly transferred to potted soil after co-cultivation, omitting the step of germinating in the presence of cefotaxime. For wheat and rice transformation, 200 µM acetosyringone was added to the bacterial suspension during co-cultivation. The co-cultivation duration was also varied from 30 min to overnight in the rice transformation experiment to check the minimum duration required for transgene transfer. We observed that 30 min of co-cultivation led to transgene transfer. Both husked and non-husked rice seeds were used for transformation and the transformation was also observed in husked seeds.

We also tested the protocol in okra, soybean and gladiolus. Seeds of these crops (bulbs in case of gladiolus) were transformed using LBA4404 harboring the pCAMBIA 1301-OsRuvB gene. To assess the transformation efficiency, seedlings were screened by amplifying genomic DNA using OsRuvB gene-specific primers. We also transformed upland cotton var. H-1098 using the *A. tumefaciens* strain EHA105 harboring pBinAR: CaMV 35S: *cry1AcF*: Ocs.

### 3.5. Screening of Putative Transgenic Plants for Presence of the Transgene

The presence of the transgene was detected either by amplifying isolated genomic DNA by PCR using transgene-specific primers or by histochemical GUS assay. The percent transformation efficiency was calculated as (number of PCR-positive or GUS stain-positive/total number of transformants tested) × 100.

**Figure 1:**
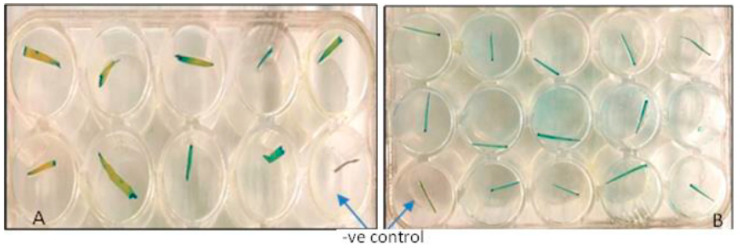
(**A**) Wheat samples showing histochemical GUS staining. (**B**) Rice samples showing histochemical GUS staining.

In the experiments listed at no. 11 and 12 in Table 2, leaf samples were taken from both rice and wheat (fifteen-day-old seedlings) and used for the GUS assay for the detection of the blue color in the putative transgenic plants. The blue color confirmed the integration and expression of the introduced *gus* gene in the plant’s genome (Figure 1).

The transformation efficiency observed in all the experiments is given in Table 2. A high transformation efficiency (93.8%) was observed when the *A. tumefaciens* strain harboring pCAMBIA 1301-*OsRuv*B was used for the transformation of indica rice var. 1121 (93.8%), followed by wheat var. 1105 (58.9%). The *A. tumefaciens* strain LBA4404 harboring pBinAR-35S*cry1Ac* used for the transformation of pigeon pea showed 45.0% transformation efficiency [19]. A transformation efficiency of 40.9% was also observed with this strain in the transformation of chickpea cv. HC-1 [18].

The transformation efficiency observed in soybean, okra and gladiolus was 51.3% (19 PCR-positive/36 screened), 75.0% (27 PCR-positive/36 screened) and 100.0% (8 PCR-positive/8 screened), respectively. Of a total of 71 cotton plants screened by PCR amplification using transgene-specific primers, 25 plants were found to be PCR-positive, yielding a transformation efficiency of 35%.

### 3.6. Determination of Copy Number by Southern Hybridization and Real-Time PCR

Genomic DNA (10 µg) isolated from the transgenic lines was digested with the respective restriction endonuclease, separated on 1.0% agarose gel and transferred onto Total BLOT+ nylon membrane. The PCR-amplified fragment of the respective transgene was eluted from the gel using the QIAquick Gel Extraction Kit (Qiagen Inc., Germantown, MA, USA) and was labeled by a non-radioactive process using biotin following the manufacturer’s manual (Biotin DecalabelTM DNA Labelling Kit, Fermentas, MA, USA). The Biotin Chromogenic Detection Kit (Fermentas, Waltham, MA, USA) was used to detect hybridized biotin-labeled probes on the nylon membrane following the kit’s manual.

The genomic DNA isolated from wild-type plants was used as a negative control and plasmid DNA was used as positive control.

Table 3 contains the details of various components of Southern hybridization experiments, such as restriction endonuclease used for digesting the genomic DNA, probes used for the detection of the integrated transgene and the generation under study in various crops we developed using the protocol.

**Figure 2:**
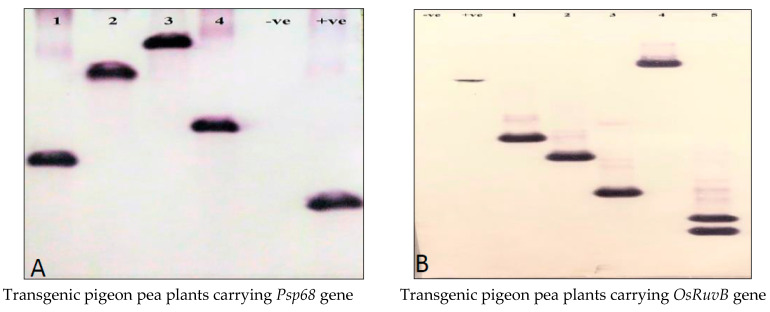

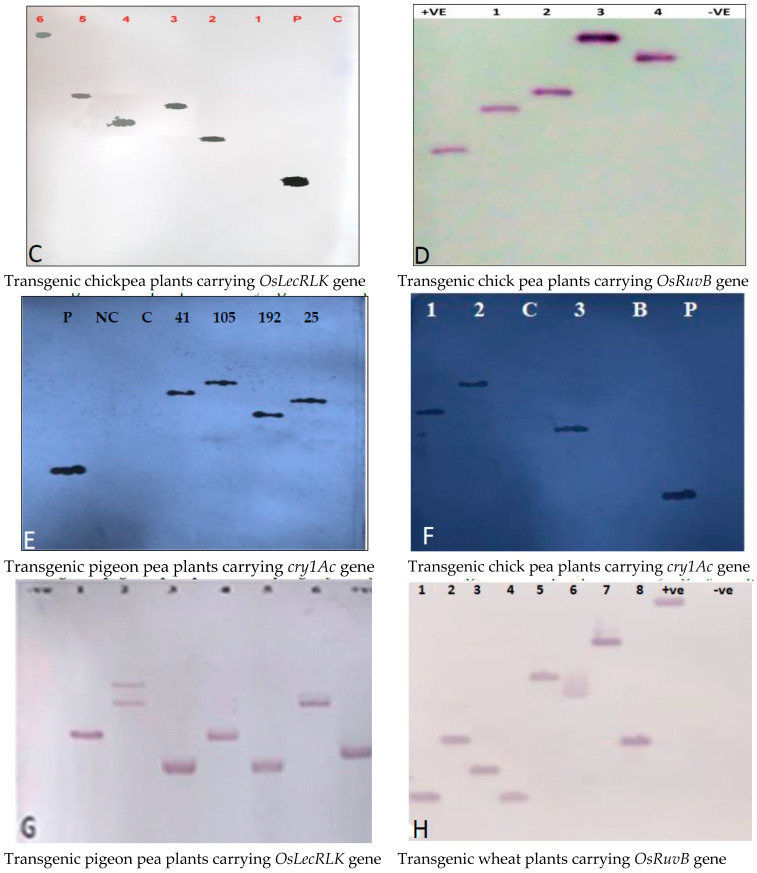
Southern blots depicting transgene integration and copy number in transgenic lines developed using the protocol [18,19,20,21,22,23,24,25].

The presence of a single band observed by Southern hybridization analysis revealed that all the transgenic lines developed in various crops reported here (Table 2) carried a single copy of the transgene (Figure 2), except transgenic pigeon pea lines separately carrying the *OsRuv*B gene and the *OsLecRLK* gene, where one of line each was observed to carry two copies (last lane in Figure 2B; third lane in Figure 2G) as two bands in each of these lines were detected. Similar results were observed by real time-PCR analysis [17,18,19,20,21,22,23,24,25,26].

### 3.7. Inheritance Pattern of Transgene in T_1_ Generation

As an example, the inheritance pattern of the OsRuvB gene in transgenic pigeon pea is presented here [20]. The inheritance pattern of the OsRuvB gene in the T_1_ generation was assessed by the presence of a transgene detected through direct PCR amplification with gene-specific primers. Segregation data were analyzed by the chi-squared test (*p* ≤ 0.05, χ^2^ = 3.841).

Segregation analysis of the transgene in T1 plants of the lines L-10, L-17, L-32, L-37 and L-66 showed segregation in a monogenic Mendelian ratio of 3:1, further confirming a single-copy insertion of the transgene, while line L-107, which had two copies of the transgene (detected by Southern hybridization, Figure 2B, last lane), was segregated in a 15:1 ratio (Table 4).

### 3.8. Evaluation of Transgene Efficacy in Transgenic Plants

#### 3.8.1. Evaluation of Transgene Efficacy for Insect Resistance in Transgenic Pigeon Pea

The Cry protein expressed by the *cry1Ac* transgene was detected to evaluate its expression in the transformants. Quantitative ELISA assay conducted on 60-day-old T_0_ transgenic pigeon pea plants using the QuantiPlate Sandwich ELISA Kit for Cry1Ab/1Ac (Envirologix, Portland, ME, USA) showed expression of Cry protein ranging from 153.5 to 562.5 ng g^−1^ FW of leaves, and in the T_1_ generation plants, the toxin protein varied from 117 to 740 ng g^−1^ FW of leaves. The transgenic lines were evaluated for insect resistance by conducting an insect bioassay by feeding first-instar larvae of *Helicoverpa armigera* (Hübner). Leaves from 20-day-old T_1_ transgenic pigeon pea plants were fed to the first instar larvae and 100% mortality was observed in five transgenic plants after 72 h [19].

#### 3.8.2. Evaluation of Transgene Efficacy for Insect Resistance in Transgenic Chick Pea Lines

Quantitative assessment of the Cry1Ac toxin by ELISA analysis of T_0_ generation transgenic chickpea plants showed the toxin in the range of 100.5 to 363.5 ng g^−1^ FW of leaves of six of each cv. C-235 and HC-1 transgenic chickpea plants. All the transgenic chickpea lines that showed appreciable levels of Cry1Ac expression (>200 ng g^−1^ FW Cry1Ac toxin) were found to exhibit phenotypic abnormalities [18].

The T_2_ generation chickpea transgenic plants expressing Cry1Ac toxins were evaluated for insecticidal activity by insect feeding bioassays performed with second-instar larvae of *H. armigera.* Larvae challenged on leaves of transgenic plants showed retarded growth after 3 days of feeding, but significant mortality was not observed [18].

**Figure 3:**
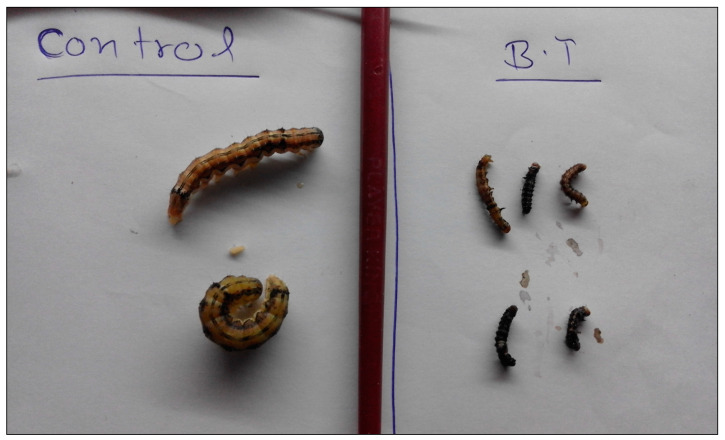
Larvae of *H. armigera* after feeding on T_3_ transgenic and non-transgenic control chick pea plants.

Prolongation in larval period and reduction in larval weight was also recorded. In T_3_ transgenic lines (Figure 3), pupation ranged from 61.6% to 100%.

#### 3.8.3. Evaluation of Transgene Efficacy for Salt Stress Tolerance in Transgenic Plants

Transgenic plants developed for salt stress tolerance (listed at 5–10, Table 2) and non-transgenic (wild-type) plants were raised in the transgenic greenhouse under pot culture conditions in dune sand soil and were subjected to NaCl stress 15 days after germination. The plants were watered with NaCl solution (100 mM in chick pea and wheat; 75 mM in pigeon pea) up to the saturation point of the soil to avoid any leaching of water. To assess the efficacy of the transgene for enhancing salt stress tolerance, various physio-biochemical parameters such as relative water content, chlorophyll content, electrolyte leakage, lipid peroxidation, proline content, total soluble sugar content, catalase and peroxidase activity were recorded 4 days and 8 days after treatment. It was observed that all the transgenic plants performed far better in comparison to wild-type plants in terms of having high chlorophyll content, relative water content, proline content, total soluble sugar content, peroxidase and catalase activity, but reduced MDA content and membrane injury index [20,21,22,23,24,25].

## 4. Discussion

In planta transformation methods involve the direct transfer of the foreign DNA to the plant tissue without an intervening step of tissue culture. Thus, it is fast and convenient method of gene transfer. A number of explant types have been reportedly used by different researchers to develop transgenic plants using this method. The most commonly used explants include apical meristem, immature embryos, inflorescence, germinating seeds, etc. There are not many reports on the use of mature seeds for transformation [28,29].

Transformation efficiency is a key point in the development of transgenic plants, and higher transformation efficiency implies the successful transfer of the transgene as well as its insertion with precision and stability. Variable *Agrobacterium*-mediated transformation efficiency is observed in different crop plants. In the current study, the *Agrobacterium* strain LBA4404 resulted in different levels of transformation efficiency in different crops. A high transformation efficiency (93.8%) in indica rice var. 1121 was observed, followed by wheat var. 1105 (58.9%). However, in wheat variety 1184, 27.0% transformation efficiency was observed with the same strain. Similar observations were recorded in chickpea varieties C-235 and HC-1, as the use of the *Agrobacterium* strain LBA4404 harboring pBinAR-35S*cry1Ac* resulted in different levels of transformation efficiency (14.3% and 40.9%). This implies that genotype also affects the transformation efficiency. In the case of *Agrobacterium*-mediated wheat transformation [30], reported the use of the *TaWOX5* gene to overcome genotype dependency. However, the regeneration step is involved in the development of transgenic wheat in their study.

A transformation efficiency of 35–41% was observed by [31] when in planta transformation of rice seed was carried out. However, the reported protocol involves pre-culturing of rice seeds for 24 h on half-strength MS medium and germination of the Agro-infected seeds on selection medium, leading to longer duration of the whole procedure. Apical meristem-targeted *Agrobacterium*-mediated transformation has been reported in cotton seedlings [32] with an initial transformation efficiency of 28–35%, followed by transferring the primary transformants to selection medium, making the protocol more time consuming. The protocol reported in the current study does not involve any pre-culturing of seeds and selection after co-cultivation. We also observed 35% transformation efficiency in upland cotton var. H-1098.

*Agrobacterium*-mediated transformation is the best choice for plant transformation due to its simple operation, high reproducibility, low copy number and low experimental cost. The ideal copy number of target genes in transgenic plants is generally one or two [33]. In all transgenic lines in various crops developed using the reported protocol, we observed single-copy insertions, except for two pigeon pea lines that separately carried two copies of the *OsRuv*B gene and the *OsLecRLK* gene. The inheritance pattern of *OsRuv*B also confirmed the transgene copy number in pigeon pea plants [20].

Our observations are in agreement with the results of other studies on *Agrobacterium-*mediated transformation, which showed that transgenic plants with low copy numbers occur more frequently than those with multiple copies. *Agrobacterium*-derived maize transformants had lower transgene copies, and higher and more stable gene expression than their bombardment-derived counterparts [34]. Similarly, all the *Agrobacterium*-derived lines integrated between one and three copies of the transgene in barley [35].

In sugarcane, the highest proportion of *Agrobacterium*-mediated transformants carried low copy numbers (estimated below two integrated copies) of the transgene [36]. Four out of six (67%) transgenic plants contained low copy numbers (one or two) in transgenic soybean developed by *Agrobacterium*-mediated transformation [37].

We have developed a tissue culture-independent transformation protocol yielding high transformation rates in different crop plants. In our protocol, no selection step is involved. Therefore, this protocol is faster than other reported protocols. We successfully developed insect-resistant chickpea and pigeon pea lines using this protocol. The Cry1Ac toxin was also found to affect *H. armigera* larval growth in the T3 generation, thereby proving the stable integration, inheritance and expression of the transgene in these lines. Similarly, salt tolerance was enhanced in pigeon pea, chickpea and wheat. Encouraging results were observed in soybean, okra and gladiolus, as well. This method demonstrates the ease of transgene transfer into the plants under study, implying its applicability in genome editing, a technique involving the transfer of desirable guide sequences for editing the target sequences. The only limitation of this method is that it cannot be used in plants that do not produce viable seeds or have vegetative modes of reproduction.

## 5. Conclusions

The in planta transformation protocol reported here was found to be robust and efficient in developing transgenic pigeon pea, chick pea and wheat. We tested the protocol in cotton, okra, rice and gladiolus and found that it works successfully with high transformation efficiency.

## Figures and Tables

**Table 1:** Troubleshooting.

S. No.	Step	Problem	Possible Reason	Solution
1	Inoculum preparation from stock culture	The culture shows no growth	The culture is not viable.	Start a new culture from a freshly plated colony.
2	Inoculum culture for further studies	Thread-like growth in the culture	The culture is contaminated.	Start the culture again and properly sterilize the inoculating needle.
3	Transformation with the gene of interest	Seeds do not germinate	Over-treatment with HgCl_2_.	Strictly follow the time and amount mentioned.
4	Isolation of genomic DNA of putative transgenic plants	Sharp and clear DNA bands are not visible	The DNA might be sheared.	Follow the given protocol without any modifications.
5	Screening of putative transgenic plants carrying the transgene	No bands or smeared patterns or multiple amplification products	The amplification did not take place or there is some problem with the primer binding.	Check for the annealing temperature based on primers and adjust it accordingly. Do not go too low to avoid non-specific binding of the primer.

**Table 2:** Details of transformation experiments conducted using the reported protocol.

Sr. No.	*Agrobacterium tumefaciens* Strain	Crop Modified	Trait	Transformation Efficiency (%)	Reference
1	EHA105 harboring pBinAR-35S*cry1Aa_3_*	Chickpea cv. C-235	Insect resistance	18.1 (6/33)	[17]
2	LBA4404 harboring pBinAR-35S*cry1Ac*	Chickpea cv. C-235	Insect resistance	14.3 (25/174)	[18]
3	LBA4404 harboring pBinAR-35S*cry1Ac*	Chickpea cv. HC-1	Insect resistance	40.9 (18/44)	[18]
4	LBA4404 harboring pBinAR-35S*cry1Ac*	Pigeon pea cv. Manak	Insect resistance	45.0 (9/20)	[19]
5	LBA4404 harboring pCAMBIA 1301-*OsRuv*B gene	Pigeon pea cv. Manak	Salt tolerance	35.7 (25/70)	[20]
6	LBA4404 harboring pCAMBIA 1301- *OsLec-RLK*	Pigeon pea cv. Manak	Salt tolerance	18.6 (16/86)	[21]
7	LBA4404 containing pCAMBIA1300 harboring *Psp68*	Pigeon pea cv. Manak	Salt tolerance	16.0 (16/100)	[22]
8	LBA4404 harboring pCAMBIA 1301-*OsRuv*B gene	Chickpea cv. HC-1	Salt tolerance	17.0 (17/100)	[23]
9	LBA4404 harboring pCAMBIA 1301-*OsLec-RLK*	Chickpea cv. HC-1	Salt tolerance	17.8 (18/101)	[24]
10	LBA4404 harboring pCAMBIA 1301-*OsRuv*B gene	Wheat var. WH1184	Salt tolerance	27.0 (26/96)	[25]
11	LBA4404 harboring pCAMBIA 1301-*OsRuv*B gene	Wheat var. 1105	--	58.9 (33/56)	[26]
12	LBA4404 harboring pCAMBIA 1301-*OsRuv*B gene	Indica rice	--	93.8 (45/49)	[26]

**Table 3:** Details of Southern hybridization analysis conducted in transgenic lines developed using the reported protocol.

S. No.	*Agrobacterium tumefaciens* Strain	Crop Modified	Generation Analyzed	Restriction Endonuclease Used for Digestion of Genomic DNA	Probe Used for Detection of the Transgene Integration	Panel in Figure 2	Reference
1	LBA4404 harboring pBinAR-35S*cry1Ac*	Chickpea cv. HC-1	T_2_	*Hind* III	PCR-amplified fragment of cry1Ac gene	Figure 2F	[18]
2	LBA4404 harboring pBinAR-35S*cry1Ac*	Pigeon pea cv. Manak	T_1_	*Hind* III	PCR-amplified fragment of cry1Ac gene	Figure 2E	[19]
3	LBA4404 harboring pCAMBIA 1301-*OsRuv*B gene	Pigeon pea cv. Manak	T_0_	*Eco*RI	PCR-amplified fragment of OsRuvB gene	Figure 2B	[20]
4	LBA4404 harboring pCAMBIA 1301- *OsLec-RLK*	Pigeon pea cv. Manak	T_0_	*Kpn*1	PCR-amplified fragment of *OsLec-RLK* gene	Figure 2G	[21]
5	LBA4404 containing pCAMBIA1300 harboring *Psp68*	Pigeon pea cv. Manak	T_0_	*Eco*RI		Figure 2A	[22]
6	LBA4404 harboring pCAMBIA 1301-*OsRuv*B gene	Chickpea cv. HC-1	T_0_	*Eco*RI	PCR-amplified fragment of OsRuvB gene	Figure 2D	[23]
7	LBA4404 harboring pCAMBIA 1301- *OsLec-RLK*	Chickpea cv. HC-1	T_1_	*Kpn*1		Figure 2C	[24]
8	LBA4404 harboring pCAMBIA 1301-*OsRuv*B gene	Wheat var. WH1184	T_0_	*Eco*RI	PCR-amplified fragment of *OsRuv*B gene	Figure 2H	[25]

**Table 4:** Segregation analyses of the transgene in T_1_ progeny of transgenic pigeon pea based on PCR analysis.

S. No.	Line	No. of T_1_ Plants Screened	*OsRuv*B +ve	*OsRuv*B −ve	Observed Ratio	χ^2^-Value	*p*-Value
1	L-10	43	31	12	2.6:1	0.21	0.64
2	L-17	42	32	10	3.2:1	0.12	0.72
3	L-32	47	34	13	2.6:1	0.10	0.75
4	L-37	50	36	14	2.6:1	0.09	0.76
5	L-66	45	33	12	2.8:1	0.11	0.74
6	L-107	36	33	3	11:1	0.52	0.47

## Data Availability

Data are available on request.

## References

[1] Alves A.C., Quecini V.M., Vieira M.L.C. (1999). Transformação de plantas: Avanços e perspectivas. Sci. Agric..

[2] Matsumoto T., Gonsalves D.K., Altman A., Hasegawa P.W. (2012). Biolistic and other non-*Agrobacterium* technologies of plant transformation. Plant Biotechnology and Agriculture.

[3] Jan S.A., Shinwari Z.K., Shah S.H., Shahzad A., Zia M.A., Ahmad N. (2016). In-planta transformation: Recent advances. Rom. Biotechnol. Lett..

[4] Feldmann K.A., Marks M.D. (1987). Agrobacterium-mediated transformation of germinating seeds of *Arabidopsis thaliana*: A non-tissue culture approach. Mol. Gen. Genet. MGG.

[5] Chang S.S., Park S.K., Kim B.C., Kang B.J., Kim D.U., Nam H.G. (1994). Stable genetic transformation of *Arabidopsis thaliana* by *Agrobacterium* inoculation in planta. Plant J..

[6] Ali A., Bang S.W., Chung S.M., Staub J.E. (2015). Plant transformation via pollen tube-mediated gene transfer. Plant Mol. Biol. Rep..

[7] Bechtold N., Bouchez D. (1995). In planta Agrobacterium-mediated transformation of adult *Arabidopsis thaliana* plants by vacuum infiltration. Gene Transfer to Plants.

[8] Kesiraju K., Sreevathsa R. (2017). Apical meristem-targeted in planta transformation strategy: An overview on its utility in crop improvement. Agric. Res. Technol. Open Access J..

[9] Niazian M., Noori S.S., Galuszka P., Mortazavian S.M.M. (2017). Tissue culture-based Agrobacterium-mediated and in planta transformation methods. Soil Water Res..

[10] Shou H., Palmer R.G., Wang K. (2002). Irreproducibility of the soybean pollen-tube pathway transformation procedure. Plant Mol. Biol. Rep..

[11] Surekha C.H., Beena M.R., Arundhati A., Singh P.K., Tuli R., Dutta-Gupta A., Kirti P.B. (2005). Agrobacterium-mediated genetic transformation of pigeon pea (*Cajanus cajan* (L.) Millsp.) using embryonal segments and development of transgenic plants for resistance against Spodoptera. Plant Sci..

[12] Sharma K.K., Sreelatha G., Dayal S. (2006). Pigeonpea (*Cajanus cajn* (L.) Millsp.). Methods Mol. Biol..

[13] Sawardekar S.V., Mhatre N.K., Sawant S.S., Bhave S.G., Gokhale N.B., Narangalkar A.L.J. (2012). Agrobacterium-mediated genetic transformation of pigeon pea [*Cajanus cajan* (L.) Millisp] for pod borer resistance: Optimization of Protocol. Indian J. Genet. Plant Bread.

[14] Parekh M.J., Mahatma M.K., Kansara R.V., Patel D.H., Jha S., Chauhan D.A. (2014). Agrobacterium Mediated Genetic Transformation of Pigeon Pea (*Cajanus cajan* L. Millsp.) using Embryonic Axes for Resistance to Lepidopteron Insect. Indian J. Agric. Biochem..

[15] Jefferson A.R. (1987). Assaying chimeric genes in plants: The GUS gene fusion system. Plant Mol. Biol. Rep..

[16] Khatodia S., Kharb P., Batra P., Chowdhury V.K. (2014). Real time PCR based detection of transgene copy number in transgenic chickpea lines expressing Cry1Aa_3_ and Cry1 Ac. Int. J. Pure App. Biosci..

[17] Khatodia S., Kharb P., Batra P., Kumar P.A., Chowdhury V.K. (2014). Molecular characterization of Bt chickpea (*Cicer arietinum* L.) plants carrying cry1Aa3 gene. Int. J. Curr. Microbiol. App. Sci..

[18] Khatodia S., Kharb P., Batra P., Chowdhury V.K. (2014). Development and characterization of transgenic chickpea (*Cicer arietinum* L.) plants with cry1Ac gene using tissue culture independent protocol. Int. J. Adv. Res..

[19] Jain M., Khatodia S., Kharb P., Batra P., Chowdhury V.K. (2017). Determination of Cry1Ac copy number in transgenic pigeonpea plants using quantitative real time PCR. Legume Res. Int. J..

[20] Singh R., Sharma S., Kharb P., Saifi S., Tuteja N. (2020). OsRuvB transgene induces salt tolerance in pigeon pea. J. Plant Interact..

[21] (2019). Pratibha. Development of Transgenic Pigeon Pea (*Cajanus cajan* L.) Plants Containing Lectin Receptor Like Kinase (Lec-RLK) Gene for Improving Salt Tolerance. Ph.D. Thesis.

[22] Kharb P.N. (2019). Psp68, A Dead Box Helicase Confers Salinity Tolerance in Transgenic PigeonPea. Int. J. Curr. Microbiol. Appl. Sci..

[23] Preeti P.K. (2020). Engineering chickpea variety HC-1 with OsRuvB gene for salt stress tolerance. Legume Res.-Int. J..

[24] Singh G. (2017). Development and Characterization of Transgenic Chickpea (*Cicer arietinum* L.) Plants with *OsLec-RLK* Gene for Salt Stress Tolerance. Ph.D. Thesis.

[25] Khasa R. (2022). Development and Characterization of Transgenic Wheat (*Triticum aestivum* L.) with *OsRuvB* Gene for Salinity Tolerance. Ph.D. Thesis.

[26] Kharb P., Singh R., Kumar U. (2019). An Efficient Method for Transformation in Monocots.

[27] Kharb P., Batra P., Chowdhury V.K. (2005). A Novel Process of Genetic Transformation in Chickpea Using Agrobacterium.

[28] Kaur R.P., Devi S. (2019). In planta transformation in plants: A review. Agric. Rev..

[29] Saifi S.K., Passricha N., Tuteja R., Pushpa K., Narendra T., Tuteja N., Tuteja R., Passricha N., Saifi S.K. (2020). In planta transformation: A smart way of crop improvement. Advancement in Crop Improvement Techniques.

[30] Wang K., Shi L., Liang X., Zhao P., Wang W., Liu J., Chang Y., Hiei Y., Yanagihara C., Du L. (2022). The gene TaWOX5 overcomes genotype dependency in wheat genetic transformation. Nat. Plants.

[31] Saifi S.K., Passricha N., Tuteja R., Nath M., Gill S.S., Tuteja N. (2021). OsRuvBL1a DNA Helicase Boost Salinity and Drought Tolerance in Transgenic Indica Rice Raised by In-Planta Transformation. Res. Sq..

[32] Kesiraju K., Mishra P., Bajpai A., Sharma M., Rao U., Sreevathsa R. (2020). *Agrobacterium tumefaciens*-mediated in planta transformation strategy for development of transgenics in cotton (*Gossypium hirsutum* L.) with GFP as a visual marker. Physiol. Mol. Biol. Plants.

[33] Tang W., Newton R.J., Weidner D.A. (2007). Genetic transformation and gene silencing mediated by multiple copies of a transgene in eastern white pine. J. Exp. Bot..

[34] Shou H., Frame B.R., Whitham S.A., Wang K. (2004). Assessment of transgenic maize events produced by particle bombardment or *Agrobacterium*-mediated transformation. Mol. Breed..

[35] Travella S., Ross S.M., Harden J., Everett C., Snape J.W., Harwood W.A. (2005). A comparison of transgenic barley lines produced by particle bombardment and *Agrobacterium*-mediated techniques. Plant Cell Rep..

[36] Mark A.J., David J.A., Robert G.B. (2013). Comparison of Agrobacterium and particle bombardment using whole plasmid or minimal cassette for production of high-expressing, low-copy transgenic plants. Transgenic Res..

[37] Li S., Cong Y., Liu Y., Wang T., Shuai Q., Chen N., Gai J., Li Y. (2017). Optimization of *Agrobacterium*-Mediated Transformation in Soybean. Front. Plant Sci..

