# Correction: Kharb et al. A Genotype-Independent, Simple, Effective and Efficient in Planta *Agrobacterium*-Mediated Genetic Transformation Protocol. *Methods Protoc.* 2022, *5*, 69

**DOI:** 10.3390/mps7020029

**Published:** 2024-03-27

**Authors:** Pushpa Kharb, Rinku Chaudhary, Narendra Tuteja, Prashant Kaushik

**Affiliations:** 1 Department of Molecular Biology, Biotechnology and Bioinformatics College of Basic Sciences and Humanities, CCSHAU, Hisar 125004, India; 2 Plant Molecular Biology Group, International Centre for Genetic Engineering & Biotechnology (ICGEB), Aruna Asaf Ali Marg, New Delhi 110067, India; 3 Kikugawa Research Station, Yokohama Ueki, 2265, Kikugawa 439-0031, Japan

Additional Affiliation(s): Updated Affiliation.

In the original publication [1], the affiliations listed for the author Prashant Kaushik were numbers 3 and 4. Affiliation 3 was: Kikugawa Research Station, Yokohama Ueki, 2265, Kikugawa 439-0031, Japan; and Affiliation 4 was: Instituto de Conservación y Mejora de la Agrodiversidad Valenciana, Universitat Politècnica de València, 46022 Valencia, Spain. Affiliation 4 has been removed per Institutional request. The authors state that the scientific conclusions are unaffected. This correction was approved by the Academic Editor. The original publication has also been updated.
